# Overview of the molecular determinants contributing to the expression of Psoriasis and Psoriatic Arthritis phenotypes

**DOI:** 10.1111/jcmm.15742

**Published:** 2020-10-31

**Authors:** Valerio Caputo, Claudia Strafella, Andrea Termine, Annunziata Dattola, Sara Mazzilli, Caterina Lanna, Terenzio Cosio, Elena Campione, Giuseppe Novelli, Emiliano Giardina, Raffaella Cascella

**Affiliations:** ^1^ Medical Genetics Laboratory Department of Biomedicine and Prevention Tor Vergata University Rome Italy; ^2^ Genomic Medicine Laboratory UILDM IRCCS Santa Lucia Foundation Rome Italy; ^3^ Dermatologic Clinic Department of Systems Medicine Tor Vergata University Rome Italy; ^4^ Neuromed Institute IRCCS Pozzilli Italy; ^5^ Department of Biomedicine and Prevention UILDM Lazio Onlus Foundation Tor Vergata University Rome Italy; ^6^ Department of Biomedical Sciences Catholic University Our Lady of Good Counsel Tirana Albania

**Keywords:** (epi)genomics, anti‐fungine response, *C. Albicans*, collagens, inflammation, keratins, Psoriasis, Psoriatic Arthritis, skin disorders

## Abstract

Psoriasis and psoriatic arthritis are multifactorial chronic disorders whose etiopathogenesis essentially derives from the alteration of several signalling pathways and the co‐occurrence of genetic, epigenetic and non‐genetic susceptibility factors that altogether affect the functional and structural property of the skin. Although shared and differential susceptibility genes and molecular pathways are known to contribute to the onset of pathological phenotypes, further research is needed to dissect the molecular causes of psoriatic disease and its progression towards Psoriatic Arthritis. This review will therefore be addressed to explore differences and similarities in the etiopathogenesis and progression of both disorders, with a particular focus on genes involved in the maintenance of the skin structure and integrity (keratins and collagens), modulation of patterns of recognition (through Toll‐like receptors and dectin‐1) and immuno‐inflammatory response (by NLRP3‐dependent inflammasome) to microbial pathogens. In addition, special emphasis will be given to the contribution of epigenetic elements (methylation pattern, non‐coding RNAs, chromatin modifiers and 3D genome organization) to the etiopathogenesis and progression of psoriasis and psoriatic arthritis. The evidence discussed in this review highlights how the knowledge of patients' clinical and (epi)genomic make‐up could be helpful for improving the available therapeutic strategies for psoriasis and psoriatic arthritis treatment.

## 1 BACKGROUND

The effectiveness of skin as protective organ is provided by a peculiar structure specifically designed to counteract a variety of dangerous events, including mechanical stress, transepidermal water loss, penetration of microorganisms or physico‐chemical irritants. Alteration in the structural properties of skin or the malfunctioning of the differentiation and maturation process among the skin layers can result in the onset of cancers or other skin‐related disorders. This kind of pathologies is very common in the general population, affecting from 30% to 70% of individuals of any age.^1^ In this context, psoriasis (Ps), psoriatic arthritis (PsA) and atopic eczema (AE) are only few examples of a wide spectrum of disease phenotypes involving the skin at different levels. Interestingly, all of them show a multifactorial aetiology characterized by the contribution of genetic and non‐genetic factors that interact together to determine a higher or lower susceptibility to develop a specific disease phenotype.^2^, ^3^, ^4^, ^5^ In particular, this review will be focused on Ps and PsA, which are both chronic disorders characterized by the dysregulation of inflammatory and immune‐mediated responses. Ps affects more than 100 million individuals worldwide. The prevalence rate is highly variable depending on the geographic area, ranging from 0.09% to 11.43%.^6^, ^7^ On this subject, the ultraviolet (UV) exposure deserves particular attention since the disease prevalence has been supposed to be correlated with the variable exposure to UV light and latitude. In fact, Ps has been found to be more frequent at higher latitude and in locations less exposed to UV light (such as in Arctic Kasach'ye displaying a prevalence of 11,8%) compared to the prevalence observed in areas closer to the equator (0% in pacific Samoa Islands).^8^ One of the possible mechanisms through which UV exposure might exert a protective role is the ability of influence the production and concentration of vitamin D. As a matter of fact, the serum levels of vitamin D have been reported to be low in psoriatic patients compared to healthy individuals in several studies.^9^ Of note, variants within genes related to vitamin D metabolism (such as *VDR*, *GC*, *CYP2R1* and *DHCR7*) have been found to interact with latitude and sun exposure, altogether contributing to the concentration of serum vitamin D levels in a cohort of Australian origin.^10^ Moreover, the higher UV exposure plays a protective role also for AE, for which the local environment plays a role in the positive/negative selection of pathogenic genetic variants.^4^ Taken together, these data suggest that UV radiations are able to interact with intrinsic factors, involving directly skin integrity and influencing Ps cutaneous manifestations development. In fact, UVB radiation influences antigen‐presenting cells (APCs), such as Langerhans cells and Langerhans cell‐like dendritic cells which, in turn, modulate the keratinocytes functions through cytokines and epidermal cell‐derived thymocyte‐activating factor (ETAF) production.^11^ In particular, the inhibition of Langerhans cells endocytic activity by UVB has been supposed to impair cell migration and maturation, suppressing the antigen‐specific immunosensitivity and, ultimately, decreasing the immuno‐inflammatory responses in the skin.^11^, ^12^ Moreover, similar mechanisms of skin photoprotection through alteration of Langerhans cells functions have been reported for UltraViolet‐Solar Simulated (UV‐SSR) and UVA radiations.^12^

**FIGURE 1:**
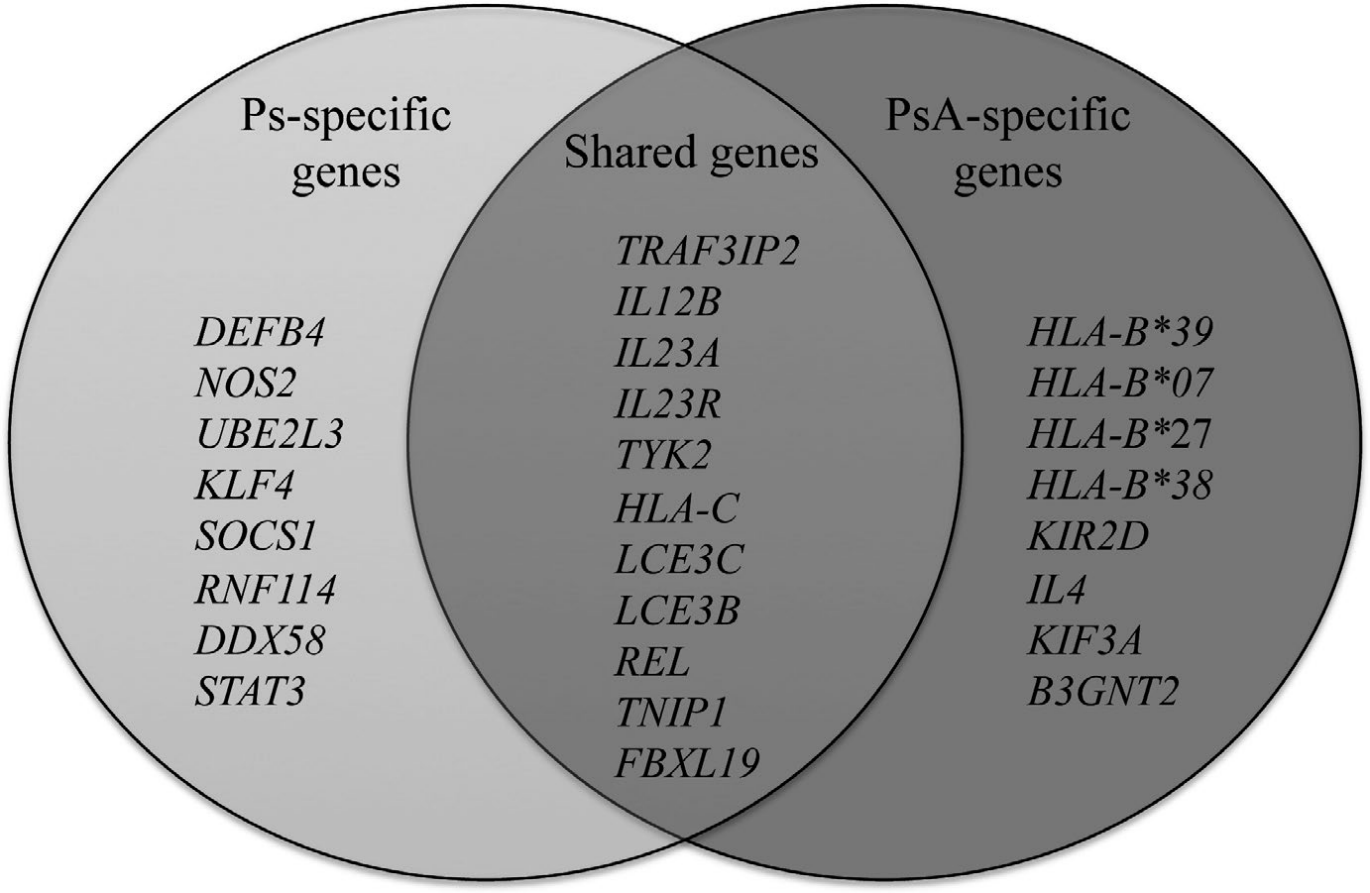
Subset of known common and differential associated genes between Psoriasis and Psoriatic Arthritis

Generally, patients affected with Ps present papulosquamous lesions (ie psoriatic plaques) which are characterized by variable morphology, distribution, severity and progression. Psoriatic plaques are usually itching, stinging and painful and are located on the scalp, elbows, knees, navel and lumbar area. Moreover, Ps can involve the oral mucosa, the soles of the feet and palms of the hands and nails. The main molecular signatures of Ps are the over‐production of cytokines that activate Th_17_ and Th_1_ cells which, in turn, trigger the amplification of inflammation and hyperproliferation of keratinocytes.^13^ Especially, the IL‐23/Th_17_/IL‐17 axis seems to play a crucial role in the initiation of the inflammatory events in Ps, whereas Th_1_/IFN‐γ and pro‐inflammatory cytokines appear to be typical of the chronic phase.^14^ Moreover, the dysfunction of regulatory T cells (Tregs) is an essential feature of Ps because of their impaired function in suppressing activation of responder T cells (Tresp) in affected patients.^15^ Approximately 20%‐30% of psoriatic patients develop PsA that affect the distal joints, the entheses and the axial skeleton over the skin. In fact, PsA patients present a dysregulation of bone morphogenesis and metabolism.^16^ The estimated prevalence of PsA ranges between 0,3% and 1%.^17^, ^18^ Although PsA and Ps show phenotypic differences, they share several susceptibility factors, especially genes mainly involved in immuno‐inflammatory responses and epidermal differentiation (Figure 1). In particular, the human leucocyte antigen (HLA)‐Cw*06:02 allele has been described as the first susceptibility factor for both Ps and PsA.^19^ Of note, HLA‐Cw*06:02 was found to mediate autoimmunity against melanocytes, through the ability of its protein product to present ADAMTS‐like protein 5 (ADAMTSL5). In fact, this complex is recognized by epidermal CD8 + T cells, which directly target melanocytes and produce inflammatory cytokines (such as TNF‐α and IL‐17) that, in turn, are able to alter melanocytes functions and proliferation, leading to dysregulation of skin homeostasis.^20^ Therefore, the alteration of melanocytes functions because of this autoimmune response could be regarded as a process by which HLA‐Cw*06:02 predispose to Ps, highlighting this disease as an autoimmune condition whose pathogenic mechanisms also depend from the individual genetic make‐up.^20^, ^21^

Several studies have identified susceptibility *HLA*‐related genes exclusively associated with PsA. In fact, the HLA‐B*39, HLA‐B*07,HLA‐B*27 and HLA‐B*38 alleles have been described as specific risk factors for PsA.^19^, ^22^ In particular, HLA‐B*27 allele has been correlated with more severe PsA phenotypes. The potential involvement of HLA‐B*39,HLA‐B*38 and HLA‐B*27 has been supported by the fact that they are able to alter the antigen recognition and binding properties, triggering thereby the activation of signalling cascades and the corresponding immune response.^19^ Moreover, the effect of the *HLA* profile on the response to treatments has been evaluated in order to identify a target therapy suitable for each patient.^23^

Over *HLA* genes, other genetic contributors have been specifically associated with PsA but not with Ps, such as *killer‐cell immunoglobulin‐like receptor* (*KIR2D* 19q13.42), *interleukin 4*, (*IL4*, 5q31.1), *kinesin family member 3A*, (*KIF3A*, 5q31.1), and *beta‐1*,*3‐N‐acetylglucosyaminyltransferase 2*, (*B3GNT2*, 2p15).^24^, ^25^, ^26^ Given the existence of common and differential susceptibility genes and molecular pathways between Ps and PsA, this review will be addressed to further explore differences and similarities in the etiopathogenesis and progression of both disorders, with a particular focus on genes involved in the maintenance of the skin structure and integrity (keratins and collagens), modulation of patterns of recognition (through Toll‐like receptors and dectin‐1) and immuno‐inflammatory response (by NLRP3‐dependent inflammasome) to microbial pathogens. In addition, special emphasis will be given to the contribution of epigenetic elements (methylation patterns, non‐coding RNAs, chromatin modifiers and 3D genome organization) to the etiopathogenesis and progression of Ps and PsA. Altogether, the investigation of genetic and epigenetic factors associated with Ps and PsA may provide additional knowledge concerning the disease mechanisms and pave the way for the development of novel diagnostic protocols and more effective treatments.

## 2 MAINTENANCE OF SKIN STRUCTURE AND INTEGRITY: THE ROLE OF KERATIN GENES

Keratins take part in cell proliferation and inflammatory response, by mediating the interactions either among cells or cells and extracellular matrix (ECM).^27^ To date, 54 Keratin (*KRT*) genes have been identified and clustered on 12q11‐q13 and 17q12‐q21 chromosomal regions. Several studies have demonstrated that *KRT1*,*KRT6A*,*KRT6B*,*KRT10*,*KRT16* and *KRT17* genes (Table 1) are aberrantly expressed in psoriatic epidermis. In particular, lower expression of KRT1 and KRT10 and higher levels of KRT6, KRT16 and KRT17 have been found in psoriatic patients, respectively.^28^, ^29^, ^30^ The involvement of *KRTs* in the etiopathogenesis of Ps is consistent with the rapid turnover, and the hyperproliferation of keratinocytes typically observed in psoriatic lesions. On this subject, the deletion of *KRT1* has been showed to result in the dysregulation of the skin barrier caused by the release of S100A8 and S100A9 heterodimers which, in turn, trigger the Toll‐like receptor 4 (TLR4)‐dependent activation of NLR family, pyrin domain‐containing 3 (NLRP3) inflammasome and, ultimately, the initiation of the inflammatory cascade.^31^ Furthermore, KRT17 has been supposed to act as an antigen, triggering an autoreactive T cell immunity response, the release of the interferon γ (IFNγ) and thereby the inflammation of epidermis.^32^ Moreover, it seems that KRT17 expression can be directly induced by IL‐17 through STAT1‐ and STAT3‐dependent mechanisms.^33^ KRT16 may activate the inflammatory response by regulating the production of glutathione (GSH) through the nuclear factor erythroid‐derived 2‐related factor 2 (NRF2) and, as a consequence, modulate the response to oxidative stress in keratinocytes.^34^ Over the role of keratins in the skin structure maintenance, highly penetrant mutations in *KRTs* can make tissues more fragile and unorganized, causing specific genetic disorders (such as epidermolytic ichthyosis, type I and II pachyonychia congenita).^27^, ^35^ However, the potential impact of *KRTs* polymorphisms on the susceptibility to Ps and PsA is still unclear and deserves to be further investigated.

**TABLE 1:** Overview of the genes potentially contributing to the etiopathogenesis of Psoriasis and Psoriatic Arthritis discussed throughout the manuscript

Gene name	Gene symbol	Genomic location
Maintenance of skin structure and integrity		
Keratin genes		
*Keratin 1*	*KRT1*	12q13.13
*Keratin 6A*	*KRT6A*	12q13.13
*Keratin 6B*	*KRT6B*	12q13.13
*Keratin 10*	*KRT10*	17q21.2
*Keratin 16*	*KRT16*	17q21.2
*Keratin 17*	*KRT17*	17q21.2
Collagen genes		
*Collagen Type I Alpha‐1*	*COL1A1*	17q21.33
*Collagen Type III Alpha‐1*	*COL3A1*	2q32.2
*Collagen Type V Alpha‐1*	*COL5A1*	9q34.3
*Collagen Type VI Alpha‐5*	*COL6A5*	3q22.1
*Collagen Type VI Alpha‐6*	*COL6A6*	3q22.1
*Collagen Type VIII Alpha‐1*,	*COL8A1*	3q12.1
*Collagen Type VIII Alpha‐2*	*COL8A2*	1p34.3
*Collagen Type II Alpha‐1*	*COL2A1*	12q13.11
*Collagen Type IX Alpha‐1*	*COL9A1*	6q13
*Collagen Type X Alpha‐1*	*COL10A1*	6q22.1
*Collagen Type XI Alpha‐1*	*COL11A1*	1p21.1
*Collagen Type XI Alpha‐2*	*COL11A2*	6p21.32
*Collagen Type XVII Alpha‐1*	*COL17A1*	10q25.1
*Collagen Type VII Alpha‐1*	*COL7A1*	3p21.31
Genes affecting responses to molecular pathogens		
*C‐type Lectin domain family 7 member A*	*CLEC7A*	12p13.2
*Toll‐Like Receptor 2*	*TLR2*	4q31.3
*Toll‐Like Receptor 3*	*TLR3*	4q35.1
*Toll‐Like Receptor 4*	*TLR4*	9q33.1
*Toll‐Like Receptor 6*	*TLR6*	4p14
*Toll‐Like Receptor 9*	*TLR9*	3p21.2
*NACHT Domain‐*,*Leucine‐Rich Repeat‐ and PYD‐containing Protein 3*	*NLRP3*	1q44
*PYD‐ and CARD domain‐containing protein*	*PYCARD*	16p11.2
*Caspase‐1*	*CASP1*	11q22.3
Genes regulating the epigenetic and transcriptional activity		
*Special AT‐rich sequence‐binding protein 1*	*SATB1*	3p24.3
*CCCTC‐Binding Factor*	*CTCF*	16q22.1
*Embryonic Ectoderm Development protein*	*EED*	11q14.2
*Enhancer of Zeste*,*Drosophila, Homolog 2*	*EZH2*	7q36.1
*Suppressor of Zeste12*	*SUZ12*	17q11.2
*MicroRNA 203a*	*MIR203A*	14q32.33
*MicroRNA 146a*	*MIR146A*	5q33.3
*MicroRNA 146b*	*MIR146B*	10q24.32
*MicroRNA 125b*	*MIR125B1*	11q24.1
*MicroRNA 21*	*MIR21*	17q23.1
*lnc‐RNU12*	*RNU12*	22q13.2
*lnc‐RP11‐701H24.7*	*RP11‐701H24.7*	15q11.2

## 3 MAINTENANCE OF SKIN STRUCTURE AND INTEGRITY: THE ROLE OF COLLAGEN GENES

Collagens are the most abundant fibrous proteins of the ECM and provide stability, resilience and strength to human tissues.^36^, ^37^ The structural properties of collagens are given by its triple‐helix conformation able to form fibrils or reticuli, which altogether allow the interaction with other ECM components and cell surface proteins. Therefore, disruption of the structure and function of collagens because of highly penetrant mutations can affect every tissue or organ and cause several pathological phenotypes. In particular, mutations within *COL17A1* cause junctional epidermolysis bullosa, non‐Herlitz‐type,^38^ whereas mutations in *COL7A1* are responsible for dominant or recessive dystrophic epidermolysis bullosa (Table 1).^39^ Furthermore, *COL1A1*, *COL3A1* and *COL5A1* (Table 1) are physiologically expressed in the skin and are involved in the proper maintenance of blood vessels. Their dysfunction, indeed, may account for vessels fragility and joint hypermobility.^40^ Other genes coding for collagen VI chains, namely *COL6A5* and *COL6A6* (Table 1), are characterized by a more limited localization in adult tissues.^41^ *COL6A5* is mainly expressed in the skin, although it has been detected in adult testis, lung and intestine.^42^ This gene has been investigated for genetic association with AE, although results are highly controversial.^43^, ^44^, ^45^, ^46^ In particular, a recent study on a cohort of 428 Mediterranean individuals (namely, Italian, Egyptian and Greek subjects) affected by AE did not find any association between *COL6A5* and AE in these populations.^47^ However, the expression of *COL6A5* in the papillary dermis^42^ suggested a potential association with Ps and PsA, given the fact that the papillary dermis can be affected in both disorders. Supporting this hypothesis, we found a significant association of rs12488457 (A/C) variant located in *COL6A5* with Ps and PsA in a recent study on the Italian population.^48^.

The investigation on *COL8A1* and *COL8A2* (Table 1) unveiled interesting insights concerning the role of ECM remodelling and angiogenesis in Ps and PsA.^49^ Previous studies found that a polymorphism (rs13081855, G/T) of *COL8A1* is associated with an increased susceptibility to exudative Age‐related macular degeneration (AMD), and it is likely to interact with the pro‐angiogenic factor VEGFA.^50^, ^51^ Concerning skin disorders, *COL8A1* has been found to be strongly associated with AE in the previously mentioned study on the Mediterranean populations.^47^ Moreover, we recently reported the association of this variant with both Ps and PsA in the Italian population, highlighting a contribution of *COL8A1* to ECM remodelling and activation of VEGFA‐related pathways in psoriatic diseases.^48^ *COL2A1*, *COL9A1*, *COL10A1*, *COL11A1* and *COL11A2* (Table 1) encode important components of cartilage and are known to play an important role in joints maintenance and bone formation.^36^, ^37^ In this context, mutations in these genes have been associated with different forms of chondrodysplasia^36^ and increased levels of collagen X have been found in the serum of PsA patients.^52^ On this subject, we found that the *COL10A1*‐rs3812111 (T/A) variant was exclusively associated with PsA, suggesting the possible contribution of bone metabolism‐related factors in the differential etiopathogenesis of PsA compared to Ps.^48^ Given these data and considering the role of collagen genes in the maintenance of bone homeostasis, their investigation may provide interesting insights into different etiopathogenetic pathways underlying Ps and PsA.^53^

## 4 GENES AFFECTING RECOGNITION PATTERNS AND IMMUNO‐INFLAMMATORY RESPONSE TO MICROBIAL PATHOGENS

The knowledge of the human microbiome represents a new standpoint in the study of complex disorders. In particular, human microbiome is characterized by bacteria, archaea, viruses and eukaryotic microbes that live in the human body. This microbial community is characterized by a variable composition, whose modifications may lead to different signatures among healthy and affected subjects. Human microbiome participates in the metabolism and interacts with the immune system and the skin barrier. In particular, skin microbial community can be highly heterogenous, although *Staphylococcus* spp., *Corynebacterium* and *Propionibacterium* represent most of bacterial populations.^54^ However, the microbial load may also be influenced by age, gender and lifestyle, suggesting that individuals may be stratified according to their microbiome profile.^55^, ^56^

The interactions between microbiome and skin are crucial for the maintenance of the barrier function, the defence from pathogens and tissue repair. On this subject, the proper maintenance of the skin homeostasis is contributed to by the production of anti‐inflammatory molecules and anti‐microbial proteins (AMPs)^54^ Moreover, the human genetic variability can also impact the interactions between microbiome and skin, leading to different immune response to the microbes in relation to the individual genetic make‐up.^58^, ^59^ The analysis of the microbiota at the level of psoriatic lesions revealed the presence of bacteria (*Staphylococcus aureus*, *Streptococcus pyogenes*), viruses (human papillomavirus‐HPV, retroviruses) and fungi (*Candida Albicans*,*Malassezia spp*.), suggesting that the onset of Ps might also be related to an excessive response to microbial pathogens.^59^ It is a matter of fact that microbial infections strongly increase the susceptibility to cutaneous disorders, especially Ps and PsA.^17^, ^60^ Interestingly, the presence of specific microorganisms on the skin of psoriatic patients may exacerbate the activation of adaptive immunity and promote the evolution towards the arthropathic form.^53^ In fact, a higher prevalence of *C Albicans* infections has been found in psoriatic patients,^61^, ^62^ The recognition mechanisms in response to fungine infections include different receptors, such as dectin‐1 and TLRs. Dectin‐1, encoded by *CLEC7A* (Table 1) is a lectin expressed on immune cells that are able to identify the saccharide wall antigens located on the surface of *C Albicans*.^62^ In vitro models showed an up‐regulation of dectin‐1 together with an alteration of Ps‐associated pathways.^19^, ^63^, ^64^ Interestingly, the stop codon polymorphism c.714T > G (rs16910526, p.Tyr238Ter) within *CLEC7A* has been found to increase the risk of developing chronic mucocutaneous candidiasis. As a matter of fact, defects in *CLEC7A* expression and activity have been found to correlate with dysregulation of inflammatory cytokines, including reduction of IL‐17, which is able to induce the production of anti‐microbial peptides. Moreover, the down‐regulation of IL17 production may prevent the treatment with IL17 inhibitors.^62^ TLRs (TLR2, TLR4, TLR6, TLR3 and TLR9) (Table 1) instead are able to recognize fungine polysaccharides and fungine nucleic acids, respectively.^65^, ^66^ The genetic association between *TLR2/TLR4* and Ps remains controversial and needs to be further investigated.^67^, ^68^, ^69^, ^70^ TLR2 is located on the surface of keratinocytes and can activate NFκB‐ and IL‐1β‐mediated pathways in response to *C Albicans* invasion.^71^, ^72^ The immune response against *C Albicans* encompasses IL‐17/IL‐23 axis, which mediates interactions between Th_17_ and Th_1_ cells.^66^ Furthermore, the presence of *C Albicans* has been shown to induce the NLRP3‐dependent inflammasome that is composed by NLRP3 (encoded by *NLRP3*) ASC (encoded by *PYCARD)* and caspase‐1 (coded by *CASP1*) (Table 1).^73^ Genetic studies reported that mutations located on *NLRP3* gene are involved in the etiopathogenesis of CINCA (chronic infantile neurologic cutaneous and articular) syndrome and familial cold auto‐inflammatory syndrome 1. In addition, *NLRP3* polymorphisms have been indicated as potential risk factors for Ps.^74^

Concerning the relationship between microbial/fungine infections and the progressive evolution towards PsA, patients showing aggressive fungine infections displayed peculiar localizations of psoriatic lesions that are nails, scalp and intergluteal cleft. As a matter of fact, nail Ps seems to be directly linked to a microbiome alteration and impairment of innate immune system. Indeed, the nail structure has a different immunological profile in Ps patients compared to healthy subjects. In fact, it is characterized by a strong innate immunity and an increased expression of anti‐microbial peptides (such as Cathelicidin‐LL37), which lead to a local production of potent immunosuppressants agents (IL‐10; TGF‐β; α‐melanocyte stimulating hormone, α‐MSH).^75^ In particular, Cathelicidin‐LL37 is activated in the nail by the release of resident microbial proteases due to the presence of one of the most frequent pathogen on the nail, namely *C Albicans*. Given this evidence, *C Albicans* could be a potential triggering factor of Ps, by activating Cathelicidin‐LL37 in the cells of the nail bed.^76^ Moreover, *C Albicans* is able to induce IL‐23 production by Th_1_ cells, thus activating Th_17_ cells. Thanks to the release of IL‐17 and IL‐22, Th_17_ cells regulate the secretion of anti‐microbial peptides (including β‐defensins) which play an essential chemotactic and pro‐inflammatory role in Ps pathogenesis. These cytokines also activate keratinocytes expressing IL‐17R, which, in turn, promote their proliferation and production of chemokines.^77^ Moreover, *C Albicans* has also been suggested as a possible triggering factor of the progression of Ps towards PsA. Of note, the psoriatic lesions in patients experiencing fungine infections have been correlated to a more severe and rapid progression of Ps towards PsA and a worse response or resistance to biological drugs. On this subject, nail involvement is more frequently observed in PsA (41%‐93%) than in Ps (15%‐50%). Several studies suggested that nail inflammation could be a specific marker of PsA because of close proximity between the nail, an enthesis (namely the distal interphalangeal joint, DIP) and the collateral ligaments around nail.^78^ However, further studies are still needed to clarify the role of microbiome and immune‐related genes (*CLEC7A*,*TLRs*, *NLRP3*, *PYCARD* and *CASP1*, Table 1) in the etiopathogenesis of Ps and its evolution towards PsA.

## 5 EPIGENETICS

Epigenetic modifications are able to induce chromatin changes without modifying the DNA sequence.^79^ Epigenetic mechanisms can operate at transcriptional (methylation and histone modifications) and post‐transcriptional (microRNAs‐miRNAs and long non‐coding RNAs‐lncRNA) level. Non‐genetic factors (including smoking habit, alcohol, stress, drugs assumption, pollution, UV radiation and diet) can induce epigenetic responses and, ultimately, modulate individual gene expression profiles and susceptibility to disease status.^80^ As a matter of fact, epigenetic modifications are known to be critically involved in the etiopathogenesis of different cutaneous complex diseases, such as Ps, AE and alopecia areata.^81^, ^82^

Concerning the contribution of DNA methylation to Ps or PsA, deregulation of the global methylation pattern has been found in psoriatic lesions.^83^, ^84^, ^85^ In particular, the promoting region of *Epidermal Differentiation Complex* (*EDC* locus, 1q21.3) genes has been found hypomethylated. This is in line with the overexpression of epidermal differentiation genes in psoriatic patients, namely *S100 calcium‐binding protein* (*S100A3*, *S100A5*, *S100A7*, *S100A12*, 1q21.3) and *late cornified envelope protein 3A* (*LCE3A*, 1q21.3).^83^ On the other hand, hypermethylation of genes involved in cell proliferation (*cyclin T1*, *CCNT1*, 12q13.11‐q13.12; *tissue inhibitor Of metalloproteinase 2*, *TIMP2*, 17q25.3) and apoptosis (*matrix metalloproteinase 9*, *MMP9*, 20q13.12; *inositol polyphosphate‐5‐phosphatase*,*145‐KD*, *INPP5D*, 2q37.1; *Annexin A4*, *ANXA4*, 2p13.3) has been reported.^84^ Altogether, these data support a role for methylation in the impairment of skin barrier function and the deregulation of keratinocyte proliferation and differentiation that is typical of Ps. However, further methylome analyses are needed to validate these data.

Chromatin regions are able to interact together by creation of *long‐range contacts* both between and within specific chromatin domains and can change throughout the cell lifetime and among different cell types. Such *long‐range chromatin contacts* allow the spatial interaction between regulatory regions (enhancers, promoters) and their target genes, contributing thereby to finely regulate gene expression, DNA replication, recombination and repair during cell development/differentiation either in physiological or in disease conditions.^86^ In this context, epigenetic modifications have been showed to influence the shaping and the rearrangements of 3D genome architecture in health and disease. Therefore, it would be interesting to understand whether specific epigenetic modifications may induce conformational changes at chromatin level which, in turn, may confer a higher/lower susceptibility to Ps or PsA. Of note, *EDC* locus has been shown to present a peculiar 3D organization in keratinocytes compared to thymocytes, where this locus is transcriptionally silent.^87^

In addition, the deregulation of chromatin remodelling genes involved in cell proliferation and differentiation has been described in subjects with Ps. On this subject, the *SATB1* gene (Table 1) acts as ‘genome organizer’ in epidermal differentiation promoting the transcription of *EDC* genes.^88^ *CTCF* gene (Table 1) codes for an insulator protein involved in the definition of boundaries among different genomic topological domains and, thus, in the control of enhancer‐promoter interactions.^86^ Moreover, Polycomb components, such as *SUZ12*, *EED* and *EZH2* (Table 1), act as transcriptional repressors in the regulation of skin development. In particular, *knock‐out* experiments demonstrated that the components of Polycomb complex 2 are important for the preservation of epidermal barrier.^89^, ^90^ In addition, Polycomb Complex 2 genes code for histone methyltransferases and signalling platforms able to recruit other epigenetic modifiers that altogether can modify the chromatin conformation. Given these data, the role of chromatin modifiers in skin disorders should be further elucidated.^91^ In addition, the genotyping analysis of genes involved in chromatin regulation might unveil potential variants that contribute to Ps and PsA susceptibility.

Over the potential contribution of chromatin remodelling and 3D genome organization, microarray and RNA‐seq approaches highlighted a possible involvement of non‐coding RNAs (ncRNAs) in Ps and PsA.^57^, ^92^ In particular, lncRNAs are involved in several biological processes, including cell development and differentiation, by interacting with other RNAs, transcription factors or chromatin‐modifying enzymes (DNA methyltransferases). LncRNAs can act as miRNAs ‘sponges’ to inhibit their activity and are able to guide both RNAs and protein factors to their sites of action. Moreover, lncRNAs can also act as scaffolds for chromosomal organization, can interfere with the post‐translational modifications between proteins and take part in signalling pathways.^93^ The characterization of deregulated lncRNAs may therefore be helpful to understand the etiopathogenesis and the progression of Ps and PsA. On this subject, a recent study by Yue et al, 2019, reported the dysregulation of 130 lncRNAs in peripheral blood mononuclear cells of PsA affected patients, suggesting a possible involvement of lncRNAs in the disease. In particular, two up‐regulated lncRNAs, namely lnc‐RP11‐701H24.7 and lnc‐RNU12 (Table 1), were significantly correlated with the disease activity and the excessive inflammatory response. According to these results, lnc‐RP11‐701H24.7 and lnc‐RNU12 were proposed as potential biomarkers for PsA.^94^

Over lncRNAs, an altered expression of miRNAs (such as miR‐203a, miR‐146a, miR‐146b, miR‐125b, miR‐21, Table 1) has also been found in psoriatic lesions and in mononuclear cells from peripheral blood of PsA affected individuals. Consistently, their target genes have also been found deregulated. Among them, it is important to mention the *suppressor of cytokine signalling 3* (*SOCS3*, 17q25.3) gene (targeted by miR‐203), which is involved in inflammatory responses in keratinocytes; and *fibroblast growth factor receptor 2*, (*FGFR2*,*10q26*.*13*, that is regulated by miR‐125), that in turn mediates skin proliferation processes. These data suggest a potential relationship between alteration of the ‘miRNaome’ and the development of a disease phenotype.^95^, ^96^, ^97^ Moreover, the genomic analysis of miRNAs genes and 3’UTR‐target genes as well as lncRNAs genes may reveal the presence of polymorphisms (SNPs, indels) that may alter the affinity binding or interaction with the expected target mRNAs and, ultimately, modify the transcriptional profile of the target genes.^95^, ^98^ On this subject, we reported the association of rs2910164 (C/G) variant within *MIR146A* sequence with both Ps and PsA in the Italian population, suggesting that the potential effect on the miRNA expression and post‐transcriptional regulation of target genes involved in skin homeostasis might contribute to the exacerbation of inflammation in Ps and PsA.^48^ Therefore, further research on variants in miRNA genes will be critical for proving the existence of a ‘genetics of epigenetics’ contributing to the onset and progression of complex disorders such as Ps and PsA.

## 6 CONCLUSIONS

**FIGURE 2:**
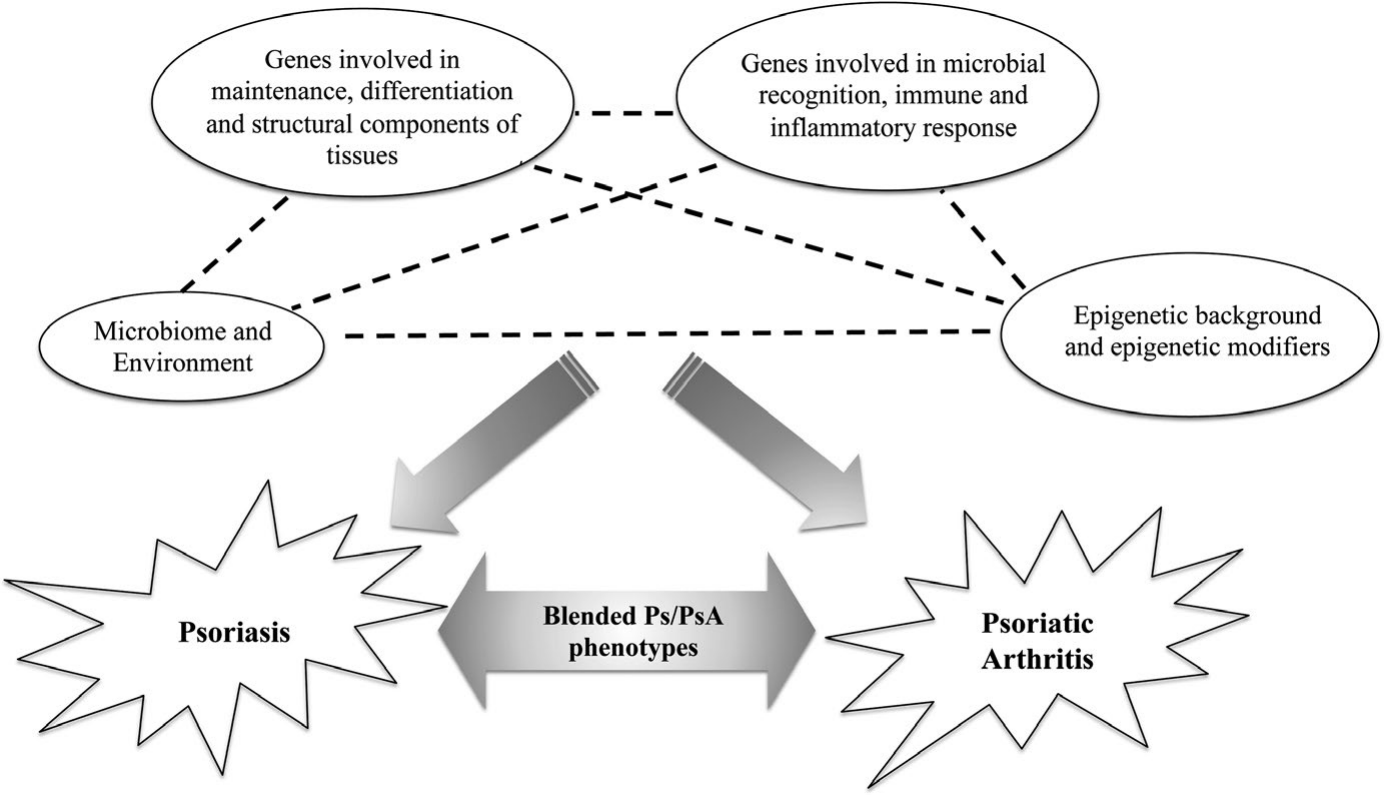
Overview of the networks underlying the etiopathogenesis of Psoriasis and Psoriatic Arthritis discussed in this manuscript. In particular, the interactions among genetic (genes coding for tissue structural components, microbial recognition and inflammatory mediators), epigenetic (epigenetic changes and epigenetic modifiers) and non‐genetic (environment and microbiome) factors contribute to the onset and progression of both the diseases and may also account for peculiar blended phenotypes (such as the severe form characterized by nail, scalp and intergluteal skin involvement)

Ps and PsA are multifactorial chronic disorders whose etiopathogenesis essentially derives from the alteration of several signalling circuits that affect the functional and structural property of the skin. In fact, the modulation of expression profiles of KRTs in keratinocytes and the consequent alteration of cell‐cell and cell‐matrix interactions (in which COLs play a fundamental role) contributes to the hyperproliferation of keratinocytes, enhancement of immuno‐inflammatory responses with over‐production of cytokines by Th_1_ and Th_17_ cells, which, ultimately, lead to the dysregulation of epidermis homeostasis. In this context, microbial agents (such as *C Albicans*) can even exacerbate skin dysfunction, perturbating the physiological activity of immune‐related factors, including the TLRs and the NLRP3‐dependent inflammasome. Therefore, the co‐occurrence of genetic, epigenetic and non‐genetic factors could explain specific skin phenotypes and the differential susceptibility to Ps and PsA (Figure 2). As a matter of fact, several studies highlighted the presence of common and different features between Ps and PsA. In particular, the existence of overlapping associated variants and biological pathways may be exploited for a drug repositioning approach, consisting of the utilization of existing drugs for the treatment of Ps and PsA patients. Such a treatment approach proved to be much more cost‐effective than designing drugs ab initio. Conversely, the knowledge of differential processes discriminating Ps from PsA is critical to better understand the specific hallmarks of disease and improve the diagnosis and the selection of the optimal therapy. In conclusion, the genetic, epigenetic and molecular factors discussed in this review may provide interesting insights concerning the biological processes underlying the etiopathogenesis of Ps and PsA, highlighting possible therapeutical targets and candidate biomarkers to be used for diagnostic and prognostic purposes. On this subject, the genes (Table 1) discussed in this review may be included in NGS panels addressed to improve the knowledge, the diagnosis and treatment of Ps and PsA. Moreover, such a gene panel may be implemented with genes involved in other dermatological and rheumatological conditions (including AE, alopecia areata, rheumatoid arthritis and idiopathic juvenile arthritis), enabling the simultaneous testing of these autoimmune disorders and facilitating the differential diagnosis. Indeed, the future of the research on Ps and PsA relies on the integration of large‐scale epigenomic data, such as information on DNA methylation, histone modifications, ncRNAs and 3D genome organization with genomic, transcriptomic, metabolomics, proteomic data and microbiomic assessment in order to achieve a deeper knowledge of the etiopathogenesis of these complex disorders. This knowledge may be then translated into the clinical practice, enabling the set‐up of precision treatment protocols which should be finalized to optimize the therapeutical strategies on the basis of patients’ clinical and (epi)genetic features^99^, ^100^ improve the patient's quality of life and prevent as much as possible harmful complications.

## CONFLICT OF INTEREST

The authors confirm that there are no conflict of interest.

## AUTHORS' CONTRIBUTIONS

**Valerio Caputo:** Conceptualization (lead); Data curation (lead); Investigation (lead); Writing‐original draft (lead); Writing‐review & editing (equal). **C. Strafella:** Conceptualization (lead); Data curation (lead); Investigation (lead); Writing‐original draft (lead); Writing‐review & editing (equal). **Andrea Termine:** Conceptualization (supporting); Data curation (equal); Investigation (supporting); Writing‐original draft (equal); Writing‐review & editing (equal). **Annunziata Dattola:** Conceptualization (supporting); Data curation (equal); Investigation (supporting); Writing‐original draft (equal); Writing‐review & editing (equal). **Sara Mazzilli:** Conceptualization (supporting); Data curation (equal); Investigation (supporting); Writing‐original draft (equal); Writing‐review & editing (equal). **Caterina Lanna:** Conceptualization (supporting); Data curation (equal); Investigation (supporting); Writing‐original draft (equal); Writing‐review & editing (equal). **Terenzio Cosio:** Conceptualization (supporting); Data curation (equal); Investigation (supporting); Writing‐original draft (equal); Writing‐review & editing (equal). **Elena Campione:** Conceptualization (lead); Data curation (supporting); Investigation (equal); Project administration (lead); Supervision (lead); Writing‐original draft (supporting); Writing‐review & editing (lead). **Giuseppe Novelli:** Conceptualization (lead); Data curation (supporting); Investigation (equal); Project administration (lead); Supervision (lead); Writing‐original draft (supporting); Writing‐review & editing (lead). **E. Giardina:** Conceptualization (lead); Data curation (supporting); Investigation (lead); Project administration (lead); Supervision (lead); Writing‐original draft (supporting); Writing‐review & editing (lead). **Raffaella Cascella:** Conceptualization (lead); Data curation (supporting); Investigation (lead); Project administration (lead); Supervision (lead); Writing‐original draft (supporting); Writing‐review & editing (lead).

## Data Availability

All data and materials were included in the manuscript.

## References

[1] Hay RJ , Johns NE , Williams HC , et al. The global burden of skin disease in 2010: an analysis of the prevalence and impact of skin conditions. J Invest Dermatol. 2014;134:1527‐1534. 24166134 10.1038/jid.2013.446

[2] Bergboer JGM , Zeeuwen PLJM , Irvine AD , et al. Deletion of late cornified envelope 3B and 3C genes is not associated with atopic dermatitis. J Invest Dermatol. 2010;130:2057‐2061. 20376060 10.1038/jid.2010.88

[3] Cascella R , Foti Cuzzola V , Lepre T , et al. Full sequencing of the FLG gene in Italian patients with atopic eczema: evidence of new mutations, but lack of an association. J Invest Dermatol. 2011;131:982‐984. 21289640 10.1038/jid.2010.398

[4] Cascella R , Strafella C , Germani C , et al. FLG (filaggrin) null mutations and sunlight exposure: evidence of a correlation. J Am Acad Dermatol. 2015;73:528‐529. 26282804 10.1016/j.jaad.2015.06.022

[5] Lepre T , Cascella R , Ragazzo M , et al. Association of KIF3A, but not OVOL1 and ACTL9, with atopic eczema in Italian patients: KIF3A is a susceptibility gene for atopic eczema in the Italian population. Br J Dermatol. 2013;168:1106‐1108. 23278845 10.1111/bjd.12178

[6] Parisi R , Symmons DPM , Griffiths CEM , et al. Global epidemiology of psoriasis: a systematic review of incidence and prevalence. J Invest Dermatol. 2013;133:377‐385. 23014338 10.1038/jid.2012.339

[7] Giardina E , Capon F , De Rosa MC , et al. Characterization of the loricrin (LOR) gene as a positional candidate for the PSORS4 psoriasis susceptibility locus. Ann Hum Genet. 2004;68:639‐645. 15598222 10.1046/j.1529-8817.2004.00118.x

[8] Gutierrez E , Sanmartino C , Carrera O , et al. Psoriasis: latitude does make a difference. J Am Acad Dermatol. 2017;77:e57. 28711107 10.1016/j.jaad.2017.03.049

[9] Hambly R , Kirby B . The relevance of serum vitamin D in psoriasis: a review. Arch Dermatol Res. 2017;309:499‐517. 28674914 10.1007/s00403-017-1751-2

[10] Lucas RM , Ponsonby A‐L , Dear K , et al. Vitamin D status: multifactorial contribution of environment, genes and other factors in healthy Australian adults across a latitude gradient. J Steroid Biochem Mol Biol. 2013;136:300‐308. 23395985 10.1016/j.jsbmb.2013.01.011

[11] Mizuno K , Okamoto H , Horio T . Ultraviolet B radiation suppresses endocytosis, subsequent maturation, and migration activity of langerhans cell‐like dendritic cells. J Invest Dermatol. 2004;122:300‐306. 15009709 10.1046/j.0022-202X.2004.22206.x

[12] Seite S , Zucchi H , Moyal D , et al. Alterations in human epidermal Langerhans cells by ultraviolet radiation: quantitative and morphological study. Br J Dermatol. 2003;148:291‐299. 12588382 10.1046/j.1365-2133.2003.05112.x

[13] Guttman‐Yassky E , Krueger JG , Lebwohl MG . Systemic immune mechanisms in atopic dermatitis and psoriasis with implications for treatment. Exp Dermatol. 2018;27:409‐417. 28266782 10.1111/exd.13336

[14] Lanna C , Mancini M , Gaziano R , et al. Skin immunity and its dysregulation in psoriasis. Cell Cycle Georget. Tex. 2019;18:2581‐2589. 10.1080/15384101.2019.1653099 PMC6773242 31416396

[15] Karamehic J , Zecevic L , Resic H , et al. Immunophenotype lymphocyte of peripheral blood in patients with psoriasis. Med Arch Sarajevo Bosnia Herzeg. 2014;68:236‐238. 10.5455/medarh.2014.68.236-238 PMC4240569 25568543

[16] Coates LC , Helliwell PS . Psoriatic arthritis: state of the art review. Clin Med Lond Engl. 2017;17:65‐70. 10.7861/clinmedicine.17-1-65 PMC6297592 28148584

[17] Cascella R , Strafella C , Longo G , et al. Pharmacogenomics of multifactorial diseases: a focus on psoriatic arthritis. Pharmacogenomics. 2016;17:943‐951. 27269419 10.2217/pgs.16.20

[18] Docampo E , Giardina E , Riveira‐Muñoz E , et al. Deletion of LCE3C and LCE3B is a susceptibility factor for psoriatic arthritis: a study in Spanish and Italian populations and meta‐analysis. Arthritis Rheum. 2011;63:1860‐1865. 21400479 10.1002/art.30340

[19] O’Rielly DD , Rahman P . Genetic, epigenetic and pharmacogenetic aspects of psoriasis and psoriatic arthritis. Rheum Dis Clin North Am. 2015;41:623‐642. 26476223 10.1016/j.rdc.2015.07.002

[20] Prinz JC . Melanocytes: target cells of an HLA‐C*06:02‐restricted autoimmune response in psoriasis. J Invest Dermatol. 2017;137:2053‐2058. 28941475 10.1016/j.jid.2017.05.023

[21] Arakawa A , Siewert K , Stöhr J , et al. Melanocyte antigen triggers autoimmunity in human psoriasis. J Exp Med. 2015;212:2203‐2212. 26621454 10.1084/jem.20151093 PMC4689169

[22] Generali E , Scirè CA , Favalli EG , et al. Biomarkers in psoriatic arthritis: a systematic literature review. Expert Re. Clin Immunol. 2016;12:651‐660. 10.1586/1744666X.2016.1147954 26821681

[23] Talamonti M , D’Adamio S , Galluccio T , et al. High‐resolution HLA typing identifies a new “super responder” subgroup of HLA‐C*06:02‐positive psoriatic patients: HLA‐C*06:02/HLA‐C*04, in response to ustekinumab. J Eur Acad Dermatol Venereol (JEADV). 2019;33:e364‐e367. 31066090 10.1111/jdv.15659

[24] Chandran V , Bull SB , Pellett FJ , et al. Killer‐cell immunoglobulin‐like receptor gene polymorphisms and susceptibility to psoriatic arthritis. Rheumatol Oxf Engl. 2014;53:233‐239. 10.1093/rheumatology/ket296 24185760

[25] Aterido A , Cañete JD , Tornero J , et al. Genetic variation at the glycosaminoglycan metabolism pathway contributes to the risk of psoriatic arthritis but not psoriasis. Ann Rheum Dis. 2019;78:e214158. 30552173 10.1136/annrheumdis-2018-214158

[26] Cascella R , Strafella C , Ragazzo M , et al. KIF3A and IL‐4 are disease‐specific biomarkers for psoriatic arthritis susceptibility. Oncotarget. 2017;8:95401‐95411. 29221136 10.18632/oncotarget.20727 PMC5707030

[27] Chamcheu JC , Siddiqui IA , Syed DN , et al. Keratin gene mutations in disorders of human skin and its appendages. Arch Biochem Biophys. 2011;508:123‐137. 21176769 10.1016/j.abb.2010.12.019 PMC3142884

[28] Desmet E , Ramadhas A , Lambert J , et al. In vitro psoriasis models with focus on reconstructed skin models as promising tools in psoriasis research. Exp Biol Med Maywood NJ. 2017;242:1158‐1169. 10.1177/1535370217710637 PMC5478008 28585891

[29] Jiang M , Li B , Zhang J , et al. Vascular endothelial growth factor driving aberrant keratin expression pattern contributes to the pathogenesis of psoriasis. Exp Cell Res. 2017;360:310‐319. 28928080 10.1016/j.yexcr.2017.09.021

[30] Ogawa E , Sato Y , Minagawa A , et al. Pathogenesis of psoriasis and development of treatment. J Dermatol. 2018;45:264‐272. 29226422 10.1111/1346-8138.14139

[31] Roth W , Kumar V , Beer H‐D , et al. Keratin 1 maintains skin integrity and participates in an inflammatory network in skin through interleukin‐18. J Cell Sci. 2012;125:5269‐5279. 23132931 10.1242/jcs.116574

[32] Shen Z , Chen L , Liu Y‐F , et al. Altered keratin 17 peptide ligands inhibit in vitro proliferation of keratinocytes and T cells isolated from patients with psoriasis. J Am Acad Dermatol. 2006;54:992‐1002. 16713453 10.1016/j.jaad.2006.02.033

[33] Shi X , Jin L , Dang E , et al. IL‐17A upregulates keratin 17 expression in keratinocytes through STAT1‐ and STAT3‐dependent mechanisms. J Invest Dermatol. 2011;131:2401‐2408. 21796151 10.1038/jid.2011.222

[34] Kerns ML , Hakim JMC , Lu RG , et al. Oxidative stress and dysfunctional NRF2 underlie pachyonychia congenita phenotypes. J Cli. Invest. 2016;126:2356‐2366. 10.1172/JCI84870 PMC4887188 27183391

[35] Knöbel M , O’Toole EA , Smith FJD . Keratins and skin disease. Cell Tissue Res. 2015;360:583‐589. 25620412 10.1007/s00441-014-2105-4

[36] Jobling R , D’Souza R , Baker N , et al. The collagenopathies: review of clinical phenotypes and molecular correlations. Curr Rheumatol Rep. 2014;16:394. 24338780 10.1007/s11926-013-0394-3

[37] Arseni L , Lombardi A , Orioli D . From structure to phenotype: impact of collagen alterations on human health. Int J Mol Sci. 2018;19:1407. 10.3390/ijms19051407 PMC5983607 29738498

[38] Condrat I , He Y , Cosgarea R , et al. Junctional epidermolysis bullosa: allelic heterogeneity and mutation stratification for precision medicine. Front Med. 2018;5:363. 10.3389/fmed.2018.00363 PMC6362712 30761300

[39] Alexeev V , Salas‐Alanis JC , Palisson F , et al. Pro‐inflammatory chemokines and cytokines dominate the blister fluid molecular signature in patients with epidermolysis bullosa and affect leukocyte and stem cell migration. J Invest Dermatol. 2017;137:2298‐2308. 28736230 10.1016/j.jid.2017.07.002 PMC5651203

[40] Syx D , De Wandele I , Rombaut L , et al. Hypermobility, the Ehlers‐Danlos syndromes and chronic pain. Clin Exp Rheumatol. 2017;35(Suppl 107):116‐122. 28967365

[41] Fitzgerald J , Holden P , Hansen U . The expanded collagen VI family: new chains and new questions. Connect Tissue Res. 2013;54:345‐350. 23869615 10.3109/03008207.2013.822865 PMC5248970

[42] Sabatelli P , Gara SK , Grumati P , et al. Expression of the collagen VI α5 and α6 chains in normal human skin and in skin of patients with collagen VI‐related myopathies. J Invest Dermatol. 2011;131:99‐107. 20882040 10.1038/jid.2010.284

[43] Söderhäll C , Marenholz I , Kerscher T , et al. Variants in a novel epidermal collagen gene (COL29A1) are associated with atopic dermatitis. PLoS Biol. 2007;5:e242. 17850181 10.1371/journal.pbio.0050242 PMC1971127

[44] Castro‐Giner F , Bustamante M , Ramon González J , et al. A pooling‐based genome‐wide analysis identifies new potential candidate genes for atopy in the European Community Respiratory Health Survey (ECRHS). BMC Med Genet. 2009;10:128. 19961619 10.1186/1471-2350-10-128 PMC2797505

[45] Harazin M , Parwez Q , Petrasch‐parwez E , et al. Variation in the COL29A1 gene in German patients with atopic dermatitis, asthma and chronic obstructive pulmonary disease. J Dermatol. 2010;37:740‐742. 20649719 10.1111/j.1346-8138.2010.00923.x

[46] Naumann A , Söderhäll C , Fölster‐Holst R , et al. A comprehensive analysis of the COL29A1 gene does not support a role in eczema. J All Clin Immunol. 2011;127(1187–1194):e7. 10.1016/j.jaci.2010.12.1123 21353297

[47] Strafella C , Caputo V , Minozzi G , et al. Atopic eczema: genetic analysis of COL6A5, COL8A1, and COL10A1 in mediterranean populations. BioMed Res Int. 2019;2019:3457898. 31275967 10.1155/2019/3457898 PMC6582825

[48] Caputo V , Strafella C , Termine A , et al. RNAseq‐based prioritization revealed COL6A5, COL8A1, COL10A1 and MIR146A as common and differential susceptibility biomarkers for psoriasis and psoriatic arthritis: confirmation from genotyping analysis of 1417 Italian subjects. Int J Mol Sci. 2020;21:2740. 10.3390/ijms21082740 PMC7215451 32326527

[49] Hansen NUB , Willumsen N , Sand JMB , et al. Type VIII collagen is elevated in diseases associated with angiogenesis and vascular remodeling. Clin Biochem. 2016;49:903‐908. 27234597 10.1016/j.clinbiochem.2016.05.023

[50] Cascella R , Strafella C , Longo G , et al. Uncovering genetic and non‐genetic biomarkers specific for exudative age‐related macular degeneration: significant association of twelve variants. Oncotarget. 2018;9:7812‐7821. 29487693 10.18632/oncotarget.23241 PMC5814260

[51] Cascella R , Strafella C , Longo G , et al. Assessing individual risk for AMD with genetic counseling, family history, and genetic testing. Eye Lond. Engl. 2018;32:446‐450. 10.1038/eye.2017.192 PMC5811711 28912512

[52] Gudmann NS , Munk HL , Christensen AF , et al. Chondrocyte activity is increased in psoriatic arthritis and axial spondyloarthritis. Arthritis Res. Ther. 2016;18:141. 27306080 10.1186/s13075-016-1040-z PMC4910260

[53] Scher JU , Ogdie A , Merola JF , et al. Preventing psoriatic arthritis: focusing on patients with psoriasis at increased risk of transition. Nat Rev Rheumatol. 2019;15:153‐166. 30742092 10.1038/s41584-019-0175-0

[54] Yamazaki Y , Nakamura Y , Núñez G . Role of the microbiota in skin immunity and atopic dermatitis. Allergol Int Off J Jpn Soc Allergol. 2017;66:539‐544. 10.1016/j.alit.2017.08.004 28882556

[55] Langan EA , Griffiths C , Solbach W , et al. The role of the microbiome in psoriasis: moving from disease description to treatment selection? Br J Dermatol. 2018;178:1020‐1027. 29071712 10.1111/bjd.16081

[56] Fierer N , Lauber CL , Zhou N , et al. Forensic identification using skin bacterial communities. Proc Natl Acad Sci USA. 2010;107:6477‐6481. 20231444 10.1073/pnas.1000162107 PMC2852011

[57] Li B , Tsoi LC , Swindell WR , et al. Transcriptome analysis of psoriasis in a large case‐control sample: RNA‐seq provides insights into disease mechanisms. J Invest Dermatol. 2014;134:1828‐1838. 24441097 10.1038/jid.2014.28 PMC4057954

[58] Bakker OB , Aguirre‐Gamboa R , Sanna S , et al. Integration of multi‐omics data and deep phenotyping enables prediction of cytokine responses. Nat Immunol. 2018;19:776‐786. 29784908 10.1038/s41590-018-0121-3 PMC6022810

[59] Fry L , Baker BS , Powles AV , et al. Psoriasis is not an autoimmune disease? Exp Dermatol. 2015;24:241‐244. 25348334 10.1111/exd.12572

[60] Boehncke W‐H . Etiology and pathogenesis of Psoriasis. Rheum Dis Clin North Am. 2015;41:665‐675. 26476225 10.1016/j.rdc.2015.07.013

[61] Sepahi S , Riahi‐Zanjani B , Ghorani‐Azam A . The role of candida albicans in the pathogenesis of psoriasis vulgaris: a systematic literature review. Rev Cli. Med. 2016;3(3):122–127.

[62] Huppler AR , Bishu S , Gaffen SL . Mucocutaneous candidiasis: the IL‐17 pathway and implications for targeted immunotherapy. Arthritis Res Ther. 2012;14:217. 22838497 10.1186/ar3893 PMC3580547

[63] de Koning HD , Rodijk‐Olthuis D , van Vlijmen‐Willems IMJJ , et al. A comprehensive analysis of pattern recognition receptors in normal and inflamed human epidermis: upregulation of dectin‐1 in psoriasis. J Invest Dermatol. 2010;130:2611‐2620. 20631729 10.1038/jid.2010.196

[64] Capon F , Burden AD , Trembath RC , et al. Psoriasis and other complex trait dermatoses: from Loci to functional pathways. J Invest Dermatol. 2012;132:915‐922. 22158561 10.1038/jid.2011.395 PMC3378482

[65] Netea MG , van de Veerdonk FL , Kullberg BJ , et al. The role of NLRs and TLRs in the activation of the inflammasome. Expert Opin Biol Ther. 2008;8:1867‐1872. 18990074 10.1517/14712590802494212

[66] Kühbacher A , Burger‐Kentischer A , Rupp S . Interaction of Candida species with the skin. Microorganisms. 2017;5:32. 10.3390/microorganisms5020032 PMC5488103 28590443

[67] Akbal A , Oğuz S , Gökmen F , et al. C‐reactive protein gene and Toll‐like receptor 4 gene polymorphisms can relate to the development of psoriatic arthritis. Clin Rheumatol. 2015;34:301‐306. 24696367 10.1007/s10067-014-2581-7

[68] Shi GE , Wang T , Li S , et al. TLR2 and TLR4 polymorphisms in Southern Chinese Psoriasis Vulgaris patients. J Dermatol Sci. 2016;83:145‐147. 27155792 10.1016/j.jdermsci.2016.04.014

[69] Smith RL , Hébert HL , Massey J , et al. Association of Toll‐like receptor 4 (TLR4) with chronic plaque type psoriasis and psoriatic arthritis. Arch Dermatol Res. 2016;308:201‐205. 26830904 10.1007/s00403-016-1620-4 PMC4796327

[70] Zabłotna M , Sobjanek M , Purzycka‐Bohdan D , et al. The significance of Toll‐like receptor (TLR) 2 and 9 gene polymorphisms in psoriasis. Postepy Dermatol Alergol. 2017;34:85‐86. 28261037 10.5114/ada.2017.65628 PMC5329111

[71] Li M , Chen Q , Shen Y , et al. Candida albicans phospholipomannan triggers inflammatory responses of human keratinocytes through Toll‐like receptor 2. Exp Dermatol. 2009;18:603‐610. 19196344 10.1111/j.1600-0625.2008.00832.x

[72] Kühbacher A , Henkel H , Stevens P , et al. Central role for dermal fibroblasts in skin model protection against candida albicans. J Infect Dis. 2017;215:1742‐1752. 28368492 10.1093/infdis/jix153

[73] Place DE , Kanneganti T‐D . Recent advances in inflammasome biology. Curr Opin Immunol. 2018;50:32‐38. 29128729 10.1016/j.coi.2017.10.011 PMC5857399

[74] Carlström M , Ekman A‐K , Petersson S , et al. Genetic support for the role of the NLRP3 inflammasome in psoriasis susceptibility. Exp Dermatol. 2012;21:932‐937. 23171454 10.1111/exd.12049

[75] Ito T , Meyer KC , Ito N , et al. Immune Privilege and the Skin. In: Nickoloff BJ, Nestle FO, editors. Curr. Dir. Autoimmun., vol. 10, Basel: KARGER; 2008, pp. 27–52. 10.1159/000131412 18460879

[76] Dorschner RA , Lopez‐Garcia B , Massie J , et al. Innate immune defense of the nail unit by antimicrobial peptides. J Am Acad Dermatol. 2004;50:343‐348. 14988673 10.1016/j.jaad.2003.09.010

[77] Byun SY , Kim BR , Choi JW , et al. Severe nail fold psoriasis extending from nail psoriasis resolved with ustekinumab: suggestion of a cytokine overflow theory in the nail unit. Ann Dermatol. 2016;28:94. 26848225 10.5021/ad.2016.28.1.94 PMC4737843

[78] McGonagle D , Ash Z , Dickie L , et al. The early phase of psoriatic arthritis. Ann Rheum Dis. 2011;70:i71‐i76. 21339224 10.1136/ard.2010.144097

[79] Furrow RE , Christiansen FB , Feldman MW . Environment‐sensitive epigenetics and the heritability of complex diseases. Genetics. 2011;189:1377‐1387. 21968193 10.1534/genetics.111.131912 PMC3241426

[80] Fogel O , Richard‐Miceli C , Tost J . Epigenetic changes in chronic inflammatory diseases. Adv Protein Chem Struct Biol. 2017;106:139‐189. 28057210 10.1016/bs.apcsb.2016.09.003

[81] Zhao M , Liang G , Wu X , et al. Abnormal epigenetic modifications in peripheral blood mononuclear cells from patients with alopecia areata. Br J Dermatol. 2012;166:226‐273. 10.1111/j.1365-2133.2011.10646.x 21936853

[82] Mervis JS , McGee JS . Epigenetic therapy and dermatologic disease: moving beyond CTCL. J Dermatol Treat. 2019;30:68‐73. 10.1080/09546634.2018.1473550 29726727

[83] Roberson EDO , Liu Y , Ryan C , et al. A subset of methylated CpG sites differentiate psoriatic from normal skin. J Invest Dermatol. 2012;132:583‐592. 22071477 10.1038/jid.2011.348 PMC3568942

[84] Zhang P , Zhao M , Liang G , et al. Whole‐genome DNA methylation in skin lesions from patients with psoriasis vulgaris. J Autoimmun. 2013;41:17‐24. 23369618 10.1016/j.jaut.2013.01.001

[85] Zhou F , Shen C , Xu J , et al. Epigenome‐wide association data implicates DNA methylation‐mediated genetic risk in psoriasis. Clin Epigen. 2016;8:131. 10.1186/s13148-016-0297-z PMC5139011 27980695

[86] Bonev B , Cavalli G . Organization and function of the 3D genome. Nat Rev Genet. 2016;17:661‐678. 27739532 10.1038/nrg.2016.112

[87] Poterlowicz K , Yarker JL , Malashchuk I , et al. 5C analysis of the epidermal differentiation complex locus reveals distinct chromatin interaction networks between gene‐rich and gene‐poor TADs in skin epithelial cells. PLoS Genet. 2017;13:e1006966. 28863138 10.1371/journal.pgen.1006966 PMC5599062

[88] Fessing MY , Mardaryev AN , Gdula MR , et al. p63 regulates Satb1 to control tissue‐specific chromatin remodeling during development of the epidermis. J Cell Biol. 2011;194:825‐839. 21930775 10.1083/jcb.201101148 PMC3207288

[89] Botchkarev VA , Mardaryev AN . Repressing the keratinocyte genome: how the polycomb complex subunits operate in concert to control skin and hair follicle development. J Invest Dermatol. 2016;136:1538‐1540. 27450498 10.1016/j.jid.2016.04.026 PMC5567744

[90] Bardot ES , Valdes VJ , Zhang J , et al. Polycomb subunits Ezh1 and Ezh2 regulate the Merkel cell differentiation program in skin stem cells. EMBO J. 2013;32:1990‐2000. 23673358 10.1038/emboj.2013.110 PMC3715854

[91] Botchkarev VA . The molecular revolution in cutaneous biology: chromosomal territories, higher‐order chromatin remodeling, and the control of gene expression in keratinocytes. J Invest Dermatol. 2017;137:e93‐e99. 28411854 10.1016/j.jid.2016.04.040 PMC5567742

[92] Gudjonsson JE , Ding J , Johnston A , et al. Assessment of the psoriatic transcriptome in a large sample: additional regulated genes and comparisons with in vitro models. J Invest Dermatol. 2010;130:1829‐1840. 20220767 10.1038/jid.2010.36 PMC3128718

[93] Gupta R , Ahn R , Lai K , et al. Landscape of long noncoding RNAs in psoriatic and healthy skin. J Invest Dermatol. 2016;136:603‐609. 27015450 10.1016/j.jid.2015.12.009 PMC5546103

[94] Yue T , Ji M , Qu H , et al. Comprehensive analyses of long non‐coding RNA expression profiles by RNA sequencing and exploration of their potency as biomarkers in psoriatic arthritis patients. BMC Immunol. 2019;20:28. 31390976 10.1186/s12865-019-0297-9 PMC6686418

[95] Huang R‐Y , Li LI , Wang M‐J , et al. An exploration of the role of MicroRNAs in psoriasis: a systematic review of the literature. Medicine (Baltimore). 2015;94:e2030. 26559308 10.1097/MD.0000000000002030 PMC4912302

[96] Hermann H , Runnel T , Aab A , et al. miR‐146b probably assists miRNA‐146a in the suppression of keratinocyte proliferation and inflammatory responses in psoriasis. J Invest Dermatol. 2017;137:1945‐1954. 28595995 10.1016/j.jid.2017.05.012 PMC5977389

[97] Ciancio G , Ferracin M , Saccenti E , et al. Characterisation of peripheral blood mononuclear cell microRNA in early onset psoriatic arthritis. Clin Exp Rheumatol. 2017;35:113‐121. 27749230

[98] Pivarcsi A , Ståhle M , Sonkoly E . Genetic polymorphisms altering microRNA activity in psoriasis–a key to solve the puzzle of missing heritability? Exp Dermatol. 2014;23:620‐624. 24917490 10.1111/exd.12469

[99] Strafella C , Caputo V , Galota MR , et al. Application of precision medicine in neurodegenerative diseases. Front Neurol. 2018;9:701. 30190701 10.3389/fneur.2018.00701 PMC6115491

[100] Novelli G , Biancolella M , Latini A , et al. Precision medicine in non‐communicable diseases. High‐Throughput. 2020;9:3. 10.3390/ht9010003 PMC7151056 32046063

